# Comment on: Synthesis of New Azo Compounds Based on *N*-(4-Hydroxyphenyl)maleimide and *N*-(4-Methylphenyl)maleimide. *Molecules* 2010, 15, 7498–7508

**DOI:** 10.3390/molecules24122319

**Published:** 2019-06-23

**Authors:** John J. Morrison, Viktoria K. Brandt, Stephen G. Yeates

**Affiliations:** School of Chemistry, University of Manchester, Manchester M13 9PL, UK; Stephen.Yeates@manchester.ac.uk

**Keywords:** spectral interpretation, educational resource, refereeing resource

## Abstract

The synthesis of (*E*)-phenylazo-3-*N*-(4-hydroxyphenyl)maleimide (**1**) using a procedure previously reported in *Molecules* is deemed to be erroneous. A detailed re-investigation of the earlier work suggests that the spectral data for key intermediates and the final product, (**1**), was mis-assigned. We conclude that compound (**1**) was not synthesized, but rather an unusual ring opening reaction of the maleimide unit of the starting material, *N*-(4-hydroxyphenyl)maleimide (**2**) leading to the generation of (*Z*)-4-((4-hydroxyphenyl)amino)-4-oxobut-2-enoic acid, (**3**) was observed instead. Examination of the original experimental data reveals systematic errors in the reporting of all of the combustion microanalytical data. Overall, the present investigation suggests that errors in the interpretation of spectral data, falsification of analytical data and selective editing of experimental results raise questions over the veracity of the work presented in the original paper.

## 1. Introduction

**Figure 1:**
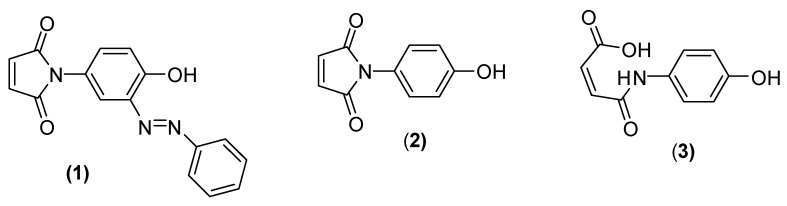
Structures of the desired product (**1**) and the two components of the mixture (**2**) and (**3**) now believed to be actual product of the reported synthesis.

We have an interest in small azobenzenes with maleimide functionality and were delighted to see the synthesis and full characterization of phenylazo-3-N-(4-hydroxyphenyl)maleimide (**1**) presented in the title paper [1] which we believed would be a useful target material for possible further thiol-ene click reactions. Unfortunately, we report that attempted preparation of (**1**) from *N*-(4-hydroxyphenyl)maleimide (**2**), using the procedure described by Mohammed and Mustapha [1], met with failure. During a thorough re-investigation of this work we note striking differences in the ^1^H-NMR spectra of (**2**) when recorded in either CDCl_3_ or CD_3_OD: In CD_3_OD maleimide (**2**) undergoes unexpected partial hydrolysis affording a mixture of (**2**) and (*Z*)-4-((4-hydroxyphenyl)amino)-4-oxobut-2-enoic acid, (**3**); maleimide (**2**) appears to be stable with respect to hydrolysis in CDCl_3_. Inexplicably, in their publication, Mohammed and Mustapha [1] assigned structure (**1**) to an equilibrating mixture of (**2**) and (**3**) in CD_3_OD. Furthermore, we suspect that the outcome of the diazo-coupling of (**2**) with diazonium salts, as reported by Mohammed and Mustapha [1], is also in error. The supposed product from one of these reactions, (*E*)-phenylazo-3-*N*-(4-hydroxyphenyl)maleimide (**1**), should now be reassigned as (*Z*)-4-((4-hydroxyphenyl)amino)-4-oxobut-2-enoic acid, (**3**) (Figure 1).

## 2. Results and Discussion

**Figure 2:**
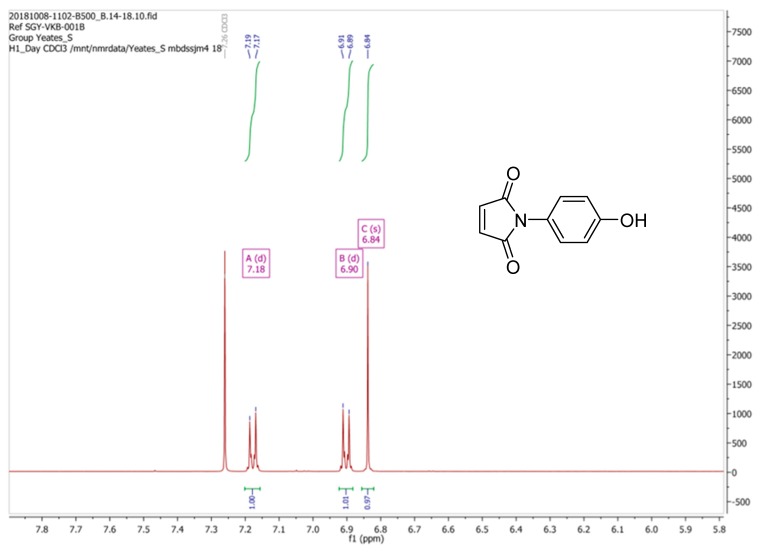
^1^H-NMR spectrum of *N*-(4-Methylphenyl)maleimide (**2**) as prepared in this study (recorded in CDCl_3_).

Reaction of *p*-aminophenol with maleic anhydride, as described by Mohammed and Mustapha [1], generated a yellow solid, albeit in a disappointingly poor yield, a result which is in accord with earlier observations. We believe that the ^1^H-NMR spectrum of (**2**), when recorded in CDCl_3_ (i.e., a 2H singlet at δ 6.83 ppm for the vinyl C-Hs of the maleimide moiety and an AB system, comprising of two, 2H doublets [δ 6.89 ppm and δ 7.17 ppm], for the aromatic hydrogens) as depicted in Figure 2 to be in keeping with its supposed structure. However, our ^1^H-NMR data was found to be substantively different to the spectrum that was reported by Mohammed and Mustapha for the same compound which was recorded in CD_3_OD. Critically these workers stated that the maleimide CHs appeared as two well-resolved doublets (centered at δ 6.79 ppm and δ 7.47 ppm) which is an assignment that is clearly improbable. In checking the literature, we note that the ^1^H-NMR spectrum for (**2**) as reported by Tang et al. [2], which was also recorded in CDCl_3_, was identical to our data (Figure 2). Being at a loss to explain these discrepancies the synthesis of (**2**) was repeated a third time using the method reported by Jin et al. [3]. Again the ^1^H-NMR spectrum (run in CDCl_3_) for this new sample of (**2**) was identical to the spectrum of the batch that we originally obtained using Mohammed and Mustapha’s procedure.

**Figure 3:**
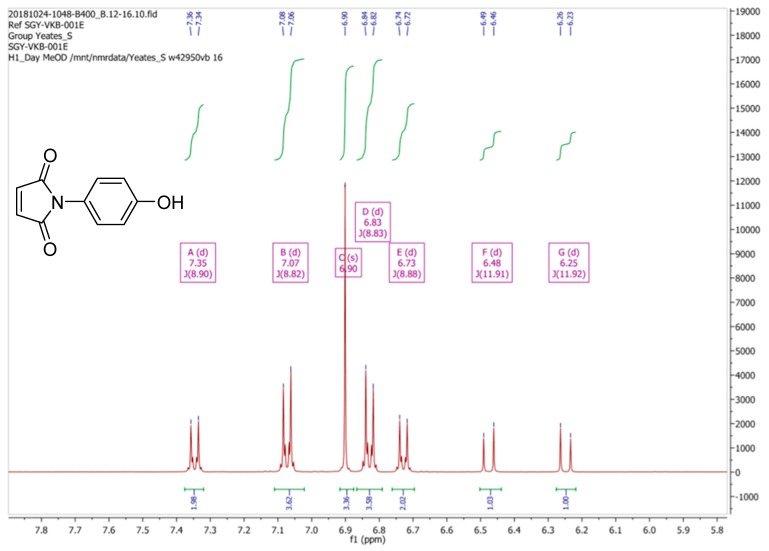
^1^H-NMR spectrum of *N*-(4-hydroxyphenyl)maleimide (**2**) recorded in CD_3_OD as prepared this study.

**Figure 4:**
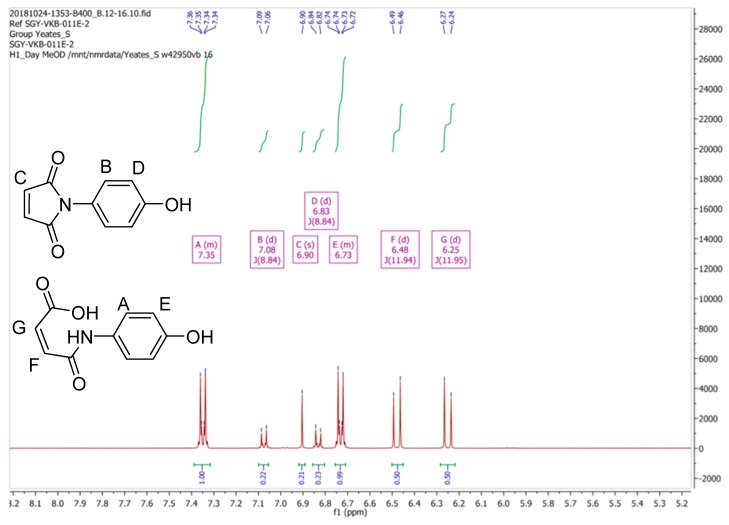
^1^H-NMR spectrum of *N*-(4-hydroxyphenyl)maleimide **2** in CD_3_OD after equilibration for 1 h.

Due to these discrepancies the ^1^H-NMR spectrum of (**2**) was then re-recorded in CD_3_OD, as originally reported by Mohammed and Mustapha [1]. Our spectral data for (**2**) in CD_3_OD is depicted in Figure 3. Clearly this spectrum is substantially different, both in terms of signal multiplicities and chemical shifts, from the spectrum that was obtained in CDCl_3_. Moreover, we observed that the ^1^H-NMR spectrum of (**2**) in CD_3_OD varied with time, a process which appeared to reach equilibrium after a period of one hour, resulting the ^1^H-NMR spectrum which is reported in Figure 4.

From the data presented in Figure 3 and Figure 4 we conclude that the resonances at δ 6.83, 6.90 and 7.08 ppm, which diminish in intensity with time, are associated with maleimide (**2**). We also suggest that a second component present in this mixture, which is associated with the resonances at δ 6.25 (1H, d, ^3^*J*11.95 Hz), 6.48 (1H, d, ^3^*J*11.94 Hz), 6.73 (2H, m) and 7.35 (2H, m) ppm, which increase in intensity with time, can be assigned to the ring-opened, (*Z*)-maleamic acid, (**3**). Furthermore, a detailed analysis of the ^1^H spectrum of (**3**), as a solution in DMSO-*d_6_*, is available in the literature [4], which is in accord with our analysis. We assume that adventitious water present in CD_3_OD is responsible for this ring opening reaction [5].

**Figure 5:**
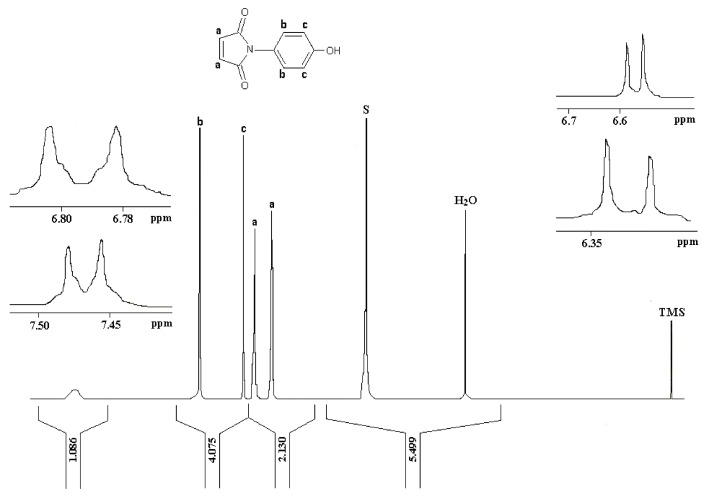
^1^H-NMR data for *N*-(4-hydroxyphenyl)maleimide (**2**) in CD_3_OD as reported by Mohammed and Mustapha [1].

At this point we note that the reporting of the NMR data for (**2**) by Mohammed and Mustapha is less than ideal. The ^1^H-NMR spectrum purported to be associated with (**2**) as reproduced in Figure 5 (doublets/multiplets at δ 6.32. 6.58, 6.79 and 7.47ppm) is at odds with that which is reported for **2** in the experimental section of the same paper [1]:

“^1^H-NMR (CD_3_OD): 7.20–7.43 (aromatic), 6.93–7.25 (HC=CH of maleimide), 6.26–6.52 (HC=CH) ppm.”

**Figure 6:**
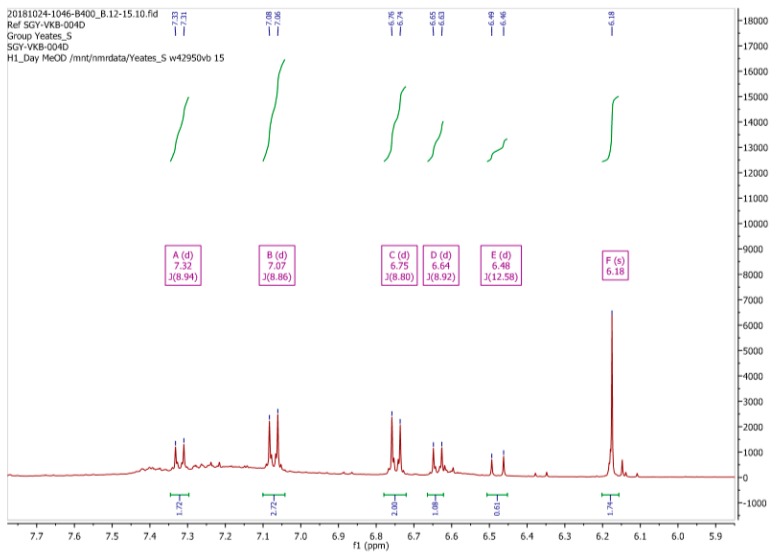
^1^H-NMR spectrum of the product from attempted preparation of (**1**) in CD_3_OD.

The proposed ring opening from (**2**) to (**3**) seems to come to an equilibrium and we have not seen it progress to full conversion. Given this and the fact that we appear to have clean material that does not show this ring-opening behavior in chloroform we decided to continue with the synthetic route reported to prepare compound (**1**). Unfortunately, despite numerous attempts this failed as the procedure detailed by Mohammed and Mustapha [1] merely afforded dark red/brown intractable solids with greater than stoichiometric yields. The most promising material resulting from these investigations afforded the proton NMR displayed in Figure 6, below. Unfortunately, GC-MS analysis of this material, which again was reported by Mohammed and Mustapha to generate a MS in accord with it structure (more on this later), proved to be less than conclusive. Indeed, we were unable to find any trace of this product by GC-MS, with the only identifiable component being due to residual (**2**) which eluted at 4.5 minutes at approximately 62 °C. We assume that other components of the mixture, as evidenced from its ^1^H-NMR spectrum, were either too volatile or in-volatile for GC-MS analysis despite allowing a long run on the instrument holding at 300 °C for 20 minutes. Again, our results appear to be at odds with those described by Mohammed and Mustapha [1]. 

**Figure 7:**
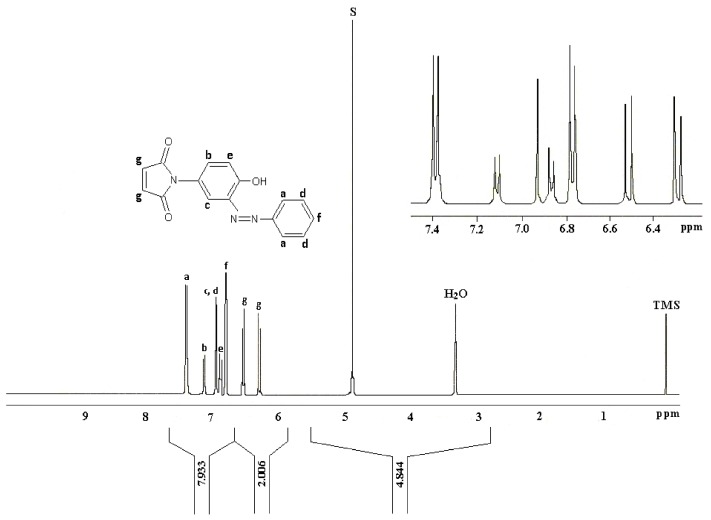
Proton NMR of phenylazo-3-*N*-(4-hydroxyphenyl)maleimide (**1**) in CD_3_OD as reported in [1].

The ^1^H-NMR data reported by Mohammed and Mustapha for (**1**) is reproduced in Figure 7. Here again this analysis was a matter of some concern especially with regards to the assignment of the maleimide CHs (“g”, Figure 7) which were reported as two doublets centered at δ 6.3 ppm and δ 6.5 ppm.

**Figure 8:**
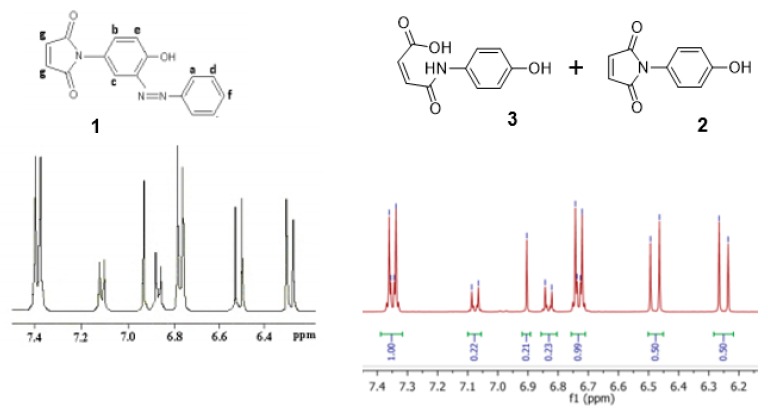
The remarkable similarity between the published ^1^H-NMR spectrum [1] for (**1**) and our ^1^H-NMR spectrum for the mixture of (**2**) and (**3**).

At this juncture we noticed a remarkable similarity between the ^1^H-NMR spectrum reported by Mohammed and Mustapha’s for azo-compound (**1**) and our ^1^H-NMR spectrum of the equilibrating mixture of (**2**) and (**2**) Figure 8. We propose that these two spectra depict the same mixture, though perhaps in different relative proportions.

**Figure 9:**
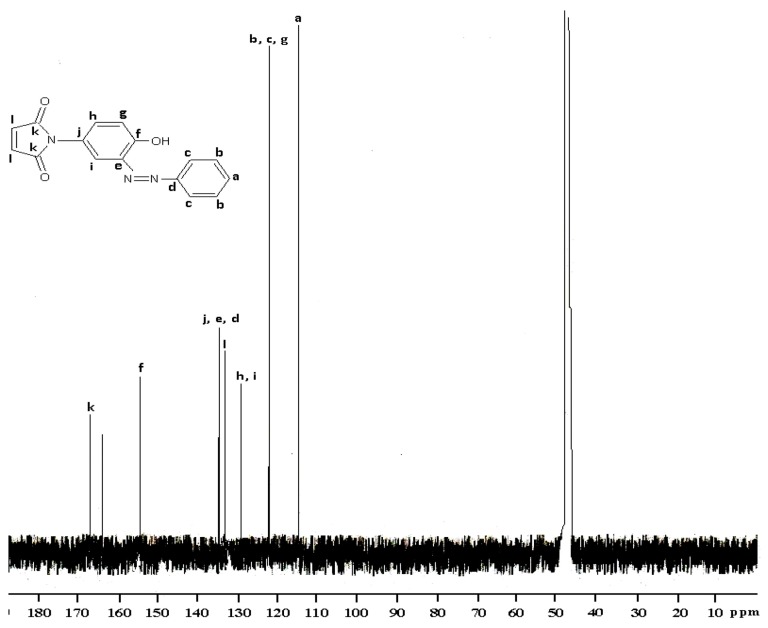
^13^C-NMR spectrum for (**1**) (in CD_3_OD) as reported by Mohammed and Mustapha [1].

We next turned our attention to the ^13^C-NMR data for (**1**) (recorded in CD_3_OD) that was reported by Mohammed and Mustapha [1], Figure 9. Here we find the assignment of what appears to be only 8 resonances for (**1**) to be erroneous. Significantly a resonance at around δ 164 ppm was simply ignored while there is an apparent accidental equivalence of three carbon signals at δ 122 ppm This is highly unusual for such a densely functionalized, low molecular weight, molecule of this type. 

**Figure 10:**
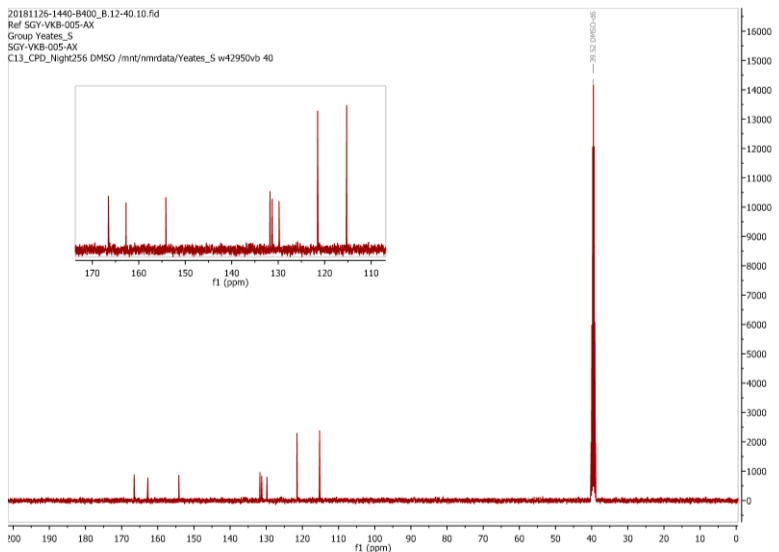
^13^C-NMR spectrum of an authentic sample of (**3**) in DMSO-*d_6_* prepared in this study.

As reported above we prepared an authentic sample of (**3**) by the method reported by Jin et al. [3] so that its NMR spectra could be recorded in DMSO-*d_6_*. In this solvent the exchangeable protons of the carboxylic acid (δ 13.71 ppm), amide (δ 10.3 ppm) and phenol (δ 9.35 ppm) residues were clearly apparent. The ^13^C-NMR spectrum of our “authentic” (**3**) in DMSO-*d_6_* (Figure 10) is also identical to that reported by Trujillo-Ferrara [4]. While we should be cognizant of potential solvent effects, we again observe a remarkable similarity between the ^13^C-NMR spectrum for (**1**) in CD_3_OD as reported by Mohammed and Mustapha and the spectrum that we observed for an authentic sample of (**3**) which was run in DMSO-*d_6_*. We contend therefore that Mohammed and Mustapha isolated (**3**) rather (**1**) from their attempted diazonium coupling reaction, a realization that is emphatically supported by the appearance of only 8 carbon signals in their reported ^13^C-NMR spectrum (Figure 9). It is now obvious that the resonances at δ 163 and δ 166 ppm reported by Mohammed and Mustapha for (**1**) are in reality associated with the two carbonyl groups (i.e., the carboxylic acid and amide functionalities) of (**3**). Awkwardly there is also a mismatch between the assignments as depicted in Figure 9 and that reported in the experimental of Mohammed and Mustapha’s original paper [1] which unexplainably does list two carbonyls but only 6 signals in total.

We also re-examined the mass spectral data for compound (**1**) as presented in Figure 3 of the original paper [1]. Although the spectrum does look authentic the molecular ion would be expected to have a mass of 293 and not of 279 as presented. It is difficult to account for how a difference of 14 can be related as a mass loss from the expected molecular ion. As reported earlier, our attempts at finding even a trace of the desired compound (**1**) by GC-MS failed but that we did find some of the starting material at *m*/*z* 189. This may also be the case for the published spectra with the possibly an impurity / column artefact at *m*/*z* 279. This is further supported by the observation that the published spectral data also displays a peak at *m*/*z* 189.

As we were only interested in synthesizing (**1**) we did not attempt to replicate any further work in the contested paper [1]. The apparent ring opening in methanol is intriguing but not what we sought [5]. The reported characterization for the other materials synthesized in [1] are somewhat difficult to follow as the proton NMR’s have no integration reported and few assignments. Most worryingly the maleimde CHs of all the products are as quoted as “multiplets”. We believe this to be erroneous. On a quick check over the number of expected ^13^C signals vs reported signals we also find far fewer reported than expected in all the diazo products reported in [1]. However, without access to the original spectroscopic data it is difficult to establish how well interpreted the written experimental for products other than (**1**) might be.

Beyond NMR interpretation all six of the azo compounds prepared in [1] are reported as having sharp melting points and in all cases excellent microanalysis results are claimed. In the case of microanalysis the greatest deviation between “found” and “expected” values is a difference of 0.18% on carbon for the phenylazo-3-*N*-(4-hydroxyphenyl)maleimide product. Unfortunately, these results must be in error as all of the calculated molecular formulas are incorrect. In the case of (**1**) the results looked very good however rather than having 23 hydrogens, (**1**) has 11. Obviously, this discrepancy of 12 H cannot possibly represent the true structure of (**1**) so we compared the results to **3** which is the best candidate from the spectroscopy presented for (**1**), Table 1.

**Figure 11:**
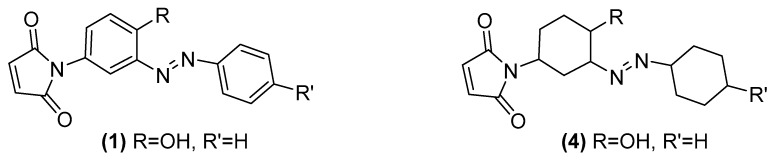
Example of possible source of error in reporting of formula for elemental analysis.

Evidently (**3**) is not a good candidate structure for the reported figures so we looked at all of the other elemental analysis published in [1]. Strangely we found that in all six of the newly reported azo compounds the same 12 hydrogen excess features in all of their published formula. We postulate that the authors may have wrongly drawn the structures as containing fully reduced, cyclohexyl, rather than aryl rings. As an example, (**4**) has an excellent match to the reported microanalysis of (**1**) and this structural alteration and its excellent fit is entirely consistent throughout the whole series of six compounds reported (Figure 11).

## 3. Experimental

### 3.1. Materials

Maleic anhydride (Sigma-Aldrich, Gillingham, UK), *p*-aminophenol (Sigma-Aldrich), sulfuric acid 98% (Fisher Chemicals, Loughborough, UK), hydrochloric acid 37% (Fisher Chemicals), sodium hydroxide (Fisher Chemicals), *N*,*N*-dimethylformamide (Sigma-Aldrich), diphosphorus pentoxide (Sigma-Aldrich), sodium nitrite (Fluka, Dresden, Germany), 2-propanol (Merck Chemicals, Loughborough, UK), chloroform-*d* (Sigma-Aldrich), dimethyl sulfoxide-*d_6_* (Cambridge Isotope Laboratories, Tewksbury, MA, USA) and methanol-*d_4_* (Cambridge Isotope Laboratories). All the chemicals were used as received without further purification.

### 3.2. Instrumentation

All NMR spectra were recorded with using a Bruker Avance III 400, Bruker Avance III HD 400 (equipped with a broadband “prodigy” N_2_ cold probe), Bruker Avance II+ 500, Bruker Avance 500 DRX or Bruker Avance III HD 500 (equipped with a broadband “prodigy” N_2_ cold probe) spectrometer (Bruker, Billerica, MA, USA). ^1^H- and ^13^C-NMR spectra were referenced to the residual solvent peak as appropriate: CDCl_3_ (7.27 or 77.00 ppm respectively), DMSO-*d*_6_ (2.50 or 39.51 ppm respectively) or methanol-*d*_4_ (3.31 or 49.15 ppm respectively). Gas chromatography (GC) mass spectra were recorded on a Perkin Elmer Auto System XL Arnel with an Agilent 5975C Triple Axis GC/MS spectrometer (Perkin Elmer, Waltham, MA, USA and Agilent Technologies, Waldbronn, Germany).

### 3.3. Repeat Synthesis of N-(4-Methylphenyl)maleimide *(**2**)*

Following the method presented in [1], starting with maleic anhydride (1.08 g, 11.0 mmol) and *p*-aminophenol (1.20 g, 11.0 mmol), 423 mg, 20% of a yellow solid was isolated. Spectroscopic analysis of this product has been presented earlier in the manuscript and is identical to that reported previously [2]. Further repetitions on larger scales with some small modifications failed to improve yields to greater than 33%. Due to the confusing nature of the NMR results we also attempted the synthesis of (**2**) using the method of Jin [3] as presented below.

4-Amino phenol (7.00 g, 64.0 mmol) was added to a solution of maleic anhydride (7.55 g, 77.0 mmol, 1.2 eq.) in 20 mL DMF at 0 °C. The mixture was stirred for 2 h and at this point a small quantity (0.2 mL) was withdrawn to provide (**3**), below. To the bulk of this reaction mixture was added, drop-wise, over a period of one hour, a solution of phosphorous pentoxide (3.71 g, 13.0 mmol) and concentrated sulfuric acid (960 µL, 18.0 mmol) in DMF (30 mL) (960 µL, 18.0 mmol). The resulting mixture was stirred at 80 °C for 5 h, cooled to room temperature and poured into 500 mL ice water. The precipitate that formed was collected at the pump, recrystallized from isopropanol and vacuum dried to afford the *title compound* (**2**), as a yellow solid. Yield 5.35 g (44%). The ^1^H-NMR spectrum of this compound is to be found in Figure 2 of this manuscript was found to be identical to that reported in [3] when run in CDCl_3_.

### 3.4. Attempted Repeat Synthesis of (E)-Phenylazo-3-N-(4-hydroxylphenyl)maleimide *(**1**)*

Using the precise procedure presented in [1] 7.88 g of a dark red/brown solid was isolated. The spectroscopic data for this product is presented earlier in the manuscript and indicated that the desired product (**1**) had not been prepared. Recrystallization of this material from glacial acetic acid was not attempted at it was clear that it did not contain any of the desired compound by GC/MS analysis. Repeating this synthesis on two separate occasions, with small alterations to work up, again failed to afford the desired product (**1**).

### 3.5. Repeat Synthesis of (Z)-4-((4-Hydroxyphenyl)amino)-4-oxobut-2-enoic Acid *(**3**)*

A small portion (0.2 mL) of the reaction mixture used in the preparation (**2**) was collected after a reaction time of 2 h. Removal of the solvent in vacuo afforded the crude (**3**). ^1^H-NMR (500 MHz, DMSO-d_6_, δ): 6.29 (d, ^3^*J* = 12.2 Hz, 1H, C*H*-COOH), 6.46 (d, ^3^*J* = 12.2 Hz, 1H, CH-CON), 6.73 (d, ^3^*J* = 8.8 Hz, 2H, Ar-H), 7.42 (d, ^3^*J* = 8.8 Hz, 2H, Ar-H), 9.35 (s, 1H, Ph-OH), 10.38 (s, 1H, NH), 13.71 (br s, 1H, COOH). This is consistent with the literature [4].

## 4. Conclusions

We have been unable to repeat the synthesis of (*E*)-phenylazo-3-*N*-(4-hydroxyphenyl)maleimide (**1**) as previously reported by Mohammed and Mustapha [1]. Examination of the published [1] procedure suggests the spectral data for (**1**) was miss-assigned and that the reported sequence actually resulted in the generation of a mixtures of *N*-(4-hydroxyphenyl)maleimide, (**2**) together with the ring-opened maleamic acid, (**3**). We have found systematic errors in the reporting of molecular formulae which strongly indicate that the microanalytical data presented in the original publication [1] was falsified. The reasons for this vitiation are unclear however this observation, in combination with the misinterpretation of spectral for (**1**), cast doubt over whether any of the six newly reported azo compounds were actually synthesized. We are trying to contact the original authors for an explanation but have received no response.

To date we note that [1] has been cited on around 20 occasions. Researchers who have used this procedure for the preparation of (**1**) and related would be best advised to make a thorough check of spectral data in order to confirm the structural identity of this and related intermediates. 

We note that, quite unexpectedly, *N*-(4-hydroxyphenyl)maleimide, (**2**) undergoes facile semi-hydrolysis in CD_3_OD to the corresponding maleamic acid, (**3**) [5].

## Figures and Tables

**Table 1:** Comparison of formula to published elemental analysis.

	Formula	Expected %	Found %
(**1**)	C_16_H_11_N_3_O_3_	C, 65.53; H, 3.78; N, 14.33	C, 62.76; H, 7.65; N, 13.86
(**3**)	C_10_H_9_NO_4_	C, 57.97; H, 4.38; N, 6.76	
(**4**)	C_16_H_23_N_3_O_3_	C, 62.92; H, 7.60; N, 13.77	
